# The Role of Cationic Polypeptides in Modulating HIV-1 Infection of the Cervicovaginal Mucosa

**DOI:** 10.3390/antibiotics3040677

**Published:** 2014-11-26

**Authors:** Amy Liese Cole, Alexander M. Cole

**Affiliations:** Burnett School of Biomedical Sciences, University of Central Florida College of Medicine, 4110 Libra Drive Building 20, Room 236, Orlando, FL 32816, USA; E-Mail: amycole@ucf.edu

**Keywords:** HIV-1, cationic, antimicrobial, peptide, protein, cervix, vagina, mucosa

## Abstract

The mucosa and overlying fluid of the female reproductive tract (FRT) are portals for the heterosexual transmission of HIV-1. Toward the ongoing development of topically applied microbicides and mucosal vaccines against HIV-1, it is evermore important to understand how the dynamic FRT mucosa is involved in controlling transmission and infection of HIV-1. Cationic peptides and proteins are the principal innate immune effector molecules of mucosal surfaces, and interact in a combinatorial fashion to modulate HIV-1 infection of the cervix and vagina. While cationic peptides and proteins have historically been categorized as antimicrobial or have other host-benefitting roles, an increasing number of these molecules have been found to augment HIV-1 infection and potentially antagonize host defense. Complex environmental factors such as hormonal fluctuations and/or bacterial and viral co-infections provide additional challenges to both experimentation and interpretation of results. In the context of heterosexual transmission of HIV-1, this review explores how various cationic peptides and proteins participate in modulating host defense against HIV-1 of the cervicovaginal mucosa.

## 1. Introduction

According to the World Health Organization (www.who.int), by the end of 2013, HIV/AIDS had claimed nearly 40 million lives. Approximately 35 million individuals worldwide were living with HIV/AIDS, and 2.1 million people were newly infected in that year alone. Nearly three-quarters (24.7 million) of infected individuals reside in sub-Saharan Africa, an area that accounts for almost 70% of the global total of new HIV infections. Since the 1980s, the spread of HIV has shifted from male-to-male sexual contact and needle sharing, to a predominantly heterosexually transmitted disease with women becoming more likely to be infected than men. One-half of infected individuals worldwide are women, a percentage that rises to nearly 60% in sub-Saharan Africa and over 75% in the young population (<25 years of age) of that region. Antiretroviral therapy (ART) is on the rise, with 12.9 million people receiving ART globally. While the numbers of individuals receiving ART are encouraging, to fully control the pandemic it will be necessary to employ multiple tactics including pre-exposure prophylaxes, which have shown promise [1], and HIV vaccines, which even after three decades of research have been terribly elusive [2]. As the field endeavors to develop these strategies, it will be evermore important to understand how the female reproductive tract (FRT) immune system is involved in controlling heterosexually transmitted HIV-1.

Considering the number of individuals infected with HIV-1, the efficiency of heterosexual HIV-1 transmission is surprisingly low. In an updated, comprehensive analysis of aggregated primary data regarding HIV transmission risk and modifying factors, Patel and colleagues estimated that the per-act HIV transmission risk for receptive uninfected females acquiring HIV-1 through penile-vaginal intercourse is 8 in 10,000 coital acts [3]. It is becoming evident that multiple physical, cellular and molecular mechanisms together contribute to keeping the incidence of transmission relatively low. The mucosal surfaces of the lower FRT, under healthy conditions are thought to act as efficient physical barriers to prevent cell-free and cell-associated HIV-1 from breaching the barrier and infecting underlying target CD4^+^ immune cells within the FRT. Indeed, the vaginal mucosa is overlain by a non-keratinized, stratified squamous epithelium approximately 150–200 microns thick on average—nearly impenetrable by 0.12 micron HIV virions unless the barrier can be subverted or compromised (e.g., abrasion, trauma). Maturation and proliferation of the vaginal epithelium is under hormonal control, with the maximum thickness occurring during time periods that normally correspond with peak circulating levels of 17β-estradiol of the late follicular phase of the menstrual cycle [4]. This would therefore suggest that times of the menstrual cycle when circulating estradiol is lowest (e.g., end of luteal phase), and thus the vaginal epithelium is thinnest, might provide a window of opportunity for HIV-1 transmission.

The lower FRT is blanketed by commensal microbes, predominantly (but not exclusively [5]) Lactobacilli in healthy individuals, which are thought to play important roles in host defense of the vagina and ectocervix. Lactobacilli render the vaginal secretions acidic by metabolizing glycogen, released by vaginal epithelia, into lactic acid that exerts selective antimicrobial activity against nonresident microbiota [6]. Certain Lactobacilli also produce hydrogen peroxide, which is toxic to many microbes at the biological concentrations measured in vaginal secretions [7]. Less advantageous microbes, such as *Gardnerella vaginalis*, are also suppressed by natural antibiotic peptides produced by Lactobacilli, called “bacteriocins” [8,9,10]. As this review is focused on human-derived antibacterial peptides and proteins secreted into vaginal fluids, it should be noted that at least a portion of the intrinsic antimicrobial activity of this fluid is of microbial origin.

Ascending the FRT, the cervix transitions to a simple columnar epithelium, the pH of the overlying fluid normalizes, and very few microbes are present in healthy individuals. Using vaginal simian immunodeficiency virus (SIV) challenge in a rhesus macaque model [11], initial cervicovaginal infection was shown to occur in small clusters of susceptible target resting and activated T lymphocytes [12]. Clusters of SIV were routinely found in two primary regions of the FRT—the endocervix, and the cervical transformation zone between the endocervix and the ectocervix [12]. These regions are located in mucosal areas of rapid cellular turnover, have a single layer of columnar epithelium, and are populated with a high density of target CD4^+^ cells, collectively providing evidence that the cervix is the primary site for initial HIV-1 infection [13]. Furthermore, from single genome amplification and sequencing of plasma virion RNA obtained from early stages of infection, it can be inferred that infection is acquired from a single founder virus in heterosexual transmission [14]. Innate processes that act as the first line of host defense against HIV-1 transmission are evermore important in preventing the establishment of this initial infection event. While other reviews and chapters have comprehensively described various aspects of innate immunity to HIV-1 infection and transmission in the FRT [15,16], this review specifically focuses on antimicrobial peptides and proteins and their role in preventing heterosexual HIV-1 infection and transmission.

## 2. Antiviral Peptides and Proteins of the FRT

Since the pioneering work of Sir Alexander Fleming in his discovery of lysozyme [17,18], and later work by James Hirsch on bactericidal histones [19], it has been known that humans have evolved various antimicrobial peptides and proteins as a first line of defense against microbial pathogens [20,21]. Most of these proteins and peptides are broad-spectrum antimicrobials, targeting gram-positive and gram-negative bacteria, fungi, and certain enveloped viruses such as HIV-1. Their mode of action can vary immensely, involving microbial membranolysis, enzymatic degradation of key microbial structural components, depletion of environmental nutrients essential for microbial growth, masking or down-regulation of receptors required for host cell entry, or modulating inflammation, adaptive immunity, and other functions related to host defense.

**Figure 1:**
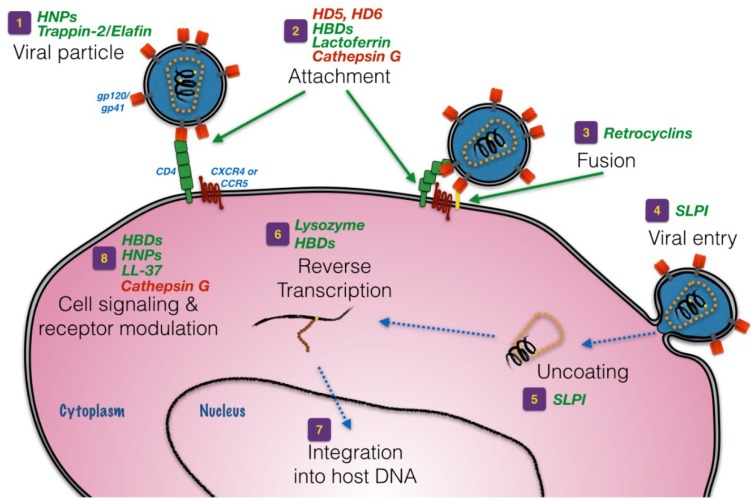
Anti-HIV-1 mechanisms of action of cationic peptides and peptides of the female reproductive tract (FRT). Depicted is the lifecycle of HIV-1 infecting a target CD4^+^ cell, beginning from a free virion (“1”) to integration of viral cDNA into genomic DNA of the target cell (“7”); “8” indicates other aspects, including receptor downmodulation and cell signaling, that indirectly affect the ability of the virus to infect or propagate within host cells. Cationic peptides and proteins in green font are antiviral at the respective stage in the lifecycle, while peptides and proteins in red font promote HIV-1 infection. Viral envelope proteins (gp120, gp41), cellular receptor (CD4) and coreceptor (CXCR4 or CCR5) required for viral attachment and entry are provided. Events downstream of viral cDNA integration into host DNA are not depicted.

Although antimicrobial peptides and proteins can be structurally and evolutionarily diverse, there are common features that encompass most classes of molecules including overall net cationic charge at physiologic pH and amphipathic separation of polar and apolar residues [22]. These basic molecular features largely contribute to the membrane-active nature of most of these cationic peptides and proteins, with the cationic side groups binding to electronegative moieties on the microbial surface and the hydrophobic groups involved in membrane penetration, pore formation and lysis/dissolution. However, as is being increasingly recognized, other modes of action cannot be entirely explained by amphipathic sequestration and/or pore formation [23,24,25,26]. The canonical lytic pore mechanism is also in contrast to the anti-HIV-1 mechanism of action of many human-derived cationic peptides and proteins. Even though some directly affect the HIV-1 virion under certain conditions [27], many others interfere with one or more specific aspects of HIV-1’s lifecycle. A number of antimicrobial peptides and proteins that are active against HIV-1 have been identified throughout the upper and lower FRT, which are discussed in the sections that follow. Importantly, while each peptide or protein has been shown to exert anti-HIV activity *in vitro* at supraphysiologic concentrations, within the cervicovaginal fluid the sum total of all of these components acting in concert is necessary for complete biological anti-HIV-1 activity [28]. Figure 1 provides a pictorial of major aspects of the HIV-1 lifecycle that are affected by cationic peptides and proteins of the FRT.

## 3. Defensins

Defensins comprise the most well-studied family of antimicrobial peptides, encompassing over 100 different peptides with a β-sheet structure, expressed by epithelia and leukocytes of many mammals and birds [29,30,31]. There are three main classes of defensins—α, β and θ—subcategorized primarily based on the disulfide bonding patterns of their six cysteine residues. In humans, α-defensins can be further divided into four peptides that are stored in neutrophil granules (human neutrophil peptides 1–4; HNP1–4), and two peptides that are inducible and mostly of epithelial origin (human defensins 5 and 6; HD5 and HD6).

HNPs are synthesized as 93- to 94-residue prepropeptides, each of which is sequentially processed to liberate a signal peptide and an anionic propiece [32,33]. The active, mature peptides are packaged principally within azurophil granules of neutrophils, where they comprise nearly 30% of the granules’ total protein content [34]. While most of the HNPs are discharged into phagocytic vacuoles where they reach millimolar concentrations, recent evidence suggests a potential extracellular arm of HNP-mediated host defense through constitutive exocytotic release of unprocessed pro-HNPs from neutrophils [35]. The first mention of the anti-HIV-1 activity of an antimicrobial peptide was reported in a short correspondence to the journal *AIDS* in 1993, whereby α-defensins from rats, guinea pigs and rabbits were shown to reduce HIV-induced cytopathogenicity of a CD4^+^ T lymphocytic cell line [36]. Monell and Strand then revealed similarities in the structure of the looped motifs from the fusogenic envelope protein gp41 of HIV-1 and α-defensins [37], pointing towards potential entry or fusion mechanisms of inhibition. Extending these findings, α-defensins were found to be directly virulytic as well as inhibit HIV-1 replication by interfering with the activity of protein kinase C [27]. In one study of healthy women, low levels (high nanogram/mL) of these α-defensins have been found in the cervicovaginal fluids [6]. As α-defensins are a marker of neutrophil influx, even low concentrations of these peptides might reveal subclinical inflammation as a constitutive host defense mechanism. Conversely, inflammation-induced recruitment of additional target cells to the area might predispose to increased susceptibility to HIV-1 infection.

Originally isolated from the Paneth cells of the small intestinal Crypts of Lieberkuhn [38,39,40], the α-defensins HD5 and HD6 are stored in secretory granules as inactive peptide precursors until extracellularly released and proteolytically activated by trypsin [41]. HD5 and HD6 have classically been categorized as broad-spectrum antimicrobials; however, these two peptides play interesting and contrasting roles in the FRT. An elegant study that comprehensively explored HD5 in the FRT determined that this peptide immunolocalized to vaginal and ectocervical epithelium, the granules within the columnar epithelium of the endocervix as well as the surface of the endocervix [42]. Even though all other human defensins have been shown to inhibit HIV-1 infection, HD5 and HD6 instead promote infection by enhancing HIV-1 attachment to target cells [43]. In this light, one might speculate whether these peptides are (co-)determinants of the cervix being the initial site for primary HIV-1 infection. HD5 expression is also modulated during the menstrual cycle, with maximal expression during the secretory phase [42]. The relatively recent theme of hormonal regulation of peptides that augment HIV-1 infection might provide unique circumstances by which HIV-1 can subvert innate antiviral defenses of the FRT.

θ-Defensins are 18 residue peptides derived from two nonapeptide precursors, which are fused in a head-to-tail fashion and rendered macrocyclic through ligation of the resulting amino and carboxyl termini [44]. Humans and nonhuman primates produce α- and β-defensin peptides; however, only select nonhuman primates produce θ-defensin peptides [44,45,46]. In humans, although θ-defensin mRNA transcripts are produced within many cells and tissues, a premature termination codon near the end of the signal sequence precludes translation. Human θ-defensin genes, called retrocyclins, are nearly 90% identical at the nucleotide level as compared to the intact rhesus macaque θ-defensin genes [44,45,46]. It is remarkable that even though retrocyclin genes are located in areas of the genome that are highly polymorphic [46,47], aside from the premature termination codon, they have been so highly conserved evolutionarily over more than 35 million years. This begs questioning the potential contemporary role of such a gene, for example whether the premature termination codon is not a true “stop”, but rather a “yield” that can be activated by an unknown molecular process. In support of this conjecture is that while under normal circumstances retrocyclin peptides have not been recovered from human cells, promyelocytes and vaginal cells and organotypic tissue constructs can be chemically coaxed to produce bioactive retrocyclins, revealing that at least the cellular machinery necessary to process these cyclic peptides remains intact in humans [48].

What we understand about retrocyclin bioactivity has occurred through the analysis of retrocyclins produced by solid-phase and other chemical syntheses. Retrocyclins are remarkably active against a broad spectrum of microbes, and were found to be particularly antiviral against herpes simplex viruses [49], influenza [50], and HIV-1 strains representing most known groups and clades [45,51,52]. Retrocyclins inhibit the ability of HIV-1 to enter target CD4^+^ cells regardless of coreceptor tropism [53], by interfering with the six-helix bundle fusogenic complex of the HIV-1 envelope glycoprotein gp41 [54]. Given its macrocyclic nature, retrocyclins are very stable peptides, which are resistant to exoproteases, a wide pH range, high temperatures (e.g., boiling, 10 min), and other degradative environments ([55,56], and A.M.C. unpublished). Due to these beneficial properties and broad activity against primary HIV-1 isolates from many worldwide clades, retrocyclins are promising topical vaginal microbicides to prevent heterosexual transmission of HIV-1 [57].

Human β-defensin (HBD) peptides are predominantly produced by epithelia and although some are constitutively expressed, many are induced by inflammatory or microbial stimuli [29,58]. HBD1–3 are expressed ubiquitously by most epithelial surfaces, while HBD4–6 appear to be more restricted to the testes and gastric antrum (HBD4) and epididymis (HBD5–6). Although HBD1 is produced at constitutively low levels throughout the body (low nanograms/mL), the highest levels of HBD1 are found in tissues of the urogenital tract, including the kidney, vagina and cervix [59], at concentrations (low-to-mid micrograms/mL) likely sufficient to contribute to antimicrobial host defense [60]. HBD1–6 are broadly active against many bacteria, fungi and viruses, and in particular HBD1–3 have been show to inhibit HIV-1 infection [61]. HBD2 and HBD3 can inhibit HIV-1 replication by down-modulating expression of CXCR4 [62], or by HBD3 antagonizing CXCR4 [63], the cellular coreceptor required for entry of X4 tropic HIV-1 into CD4^+^ cells. In vaginal fluid and cervical mucus plugs, HBD2 is present at concentrations (nanograms/mL) below the amount thought to be essential for effective direct anti-HIV-1 activity (low micrograms/mL) [6,60]. However, those concentrations are within the range that could impart other immunological functions. For example, HBD2 is a natural ligand for cells elaborating the chemokine receptor CCR6 such as a potential target of HIV-1, CD45^Ro+^/CD4^+^ T cells, as well as immature dendritic cells [64]. HBD2 and HBD3 have also been reported to chemoattract cells expressing CCR2, including macrophages, monocytes and neutrophils [65]. While unknown for cells within the FRT, as described for oral epithelial cells HIV-1 can induce the expression of HBD2 and HBD3, but not HBD1 [62]. Although β-defensins might not be directly participating in antiviral host defense, their presence and activation might attract additional cellular targets for HIV-1.

## 4. Whey Acidic Protein (WAP) Motif-Based Proteins

Secretory leukocyte protease inhibitor (SLPI) and Trappin-2/Elafin are members of the whey acidic protein (WAP) family [66,67], ascribed primary anti-inflammatory functions of inhibiting proteases including proteinase-3 and neutrophil elastase from neutrophils [68]. SLPI and Trappin-2/Elafin are secreted into overlying mucosal fluids, and both proteins exhibit antimicrobial activity (reviewed in [67]). Reports of the intrinsic anti-HIV-1 activity of SLPI have been mixed. High nanomolar concentrations of SLPI were reported to block HIV-1 entry or capsid uncoating independent of the protease inhibitor function of SLPI [69,70]. Another study suggested that the anti-HIV-1 activity of SLPI was likely due to artifact as even extremely high concentrations (1000 μg/mL) were not active against HIV-1 [71]. Evidence providing further support of an anti-HIV-1 role for SLPI has been through clinical correlative studies. Increased SLPI concentrations within vaginal fluid were associated with reduced rates of perinatal HIV-1 transmission [72], an association that was not observed for other cationic antimicrobial proteins or peptides. SLPI has also been shown to be decreased in women suffering from various sexually transmitted infections, and these reduced levels may predispose women to HIV-1 and other infections [73]. Perhaps the anti-HIV-1 activity of SLPI is best realized in concert with other endogenous antivirals.

In an elegant study, Ghosh and colleagues revealed that epithelia of the upper and lower FRT produce constitutive amounts of Trappin-2/Elafin protein and mRNA [74], further supporting findings that Trappin-2/Elafin is produced by the cervical glandular epithelium during pregnancy [75]. Interestingly, only the uterine cells of the upper FRT could upregulate Trappin-2/Elafin when stimulated with a double-stranded RNA mimic, Poly(I:C). This group further explored the direct anti-HIV roles of Trappin-2/Elafin against X4 tropic and R5 tropic HIV-1, revealing dose-dependent direct activity against HIV-1 virions [74]. Additional studies provided further support for the role of Trappin-2/Elafin in innate anti-HIV-1 host defense. CVL from HIV-negative individuals contained higher amounts of Trappin-2/Elafin than HIV-infected patients. Similar to other cationic antimicrobial peptides and proteins, Trappin-2/Elafin expression is likely under hormonal control as the concentration of this protein in CVL was significantly higher during the secretory phase of the menstrual cycle as compared to the proliferative phase [74].

## 5. Other Anti-HIV Peptides and Proteins

Cathelicidins are a family of very diverse antimicrobial peptides that each share a common amino-terminal cathelin propiece, which is similar to the thiol protease inhibitor cystatin [76]. Even though pigs, cows, and other animals contain numerous different cathelicidins, humans are endowed with only one cathelicidin called human cationic protein of 18 kDa (hCAP18) [77,78]. Depending on the cellular or histological environment, hCAP18 can be preteolytically cleaved into the mature, active forms LL-37, ALL-38, and FALL-39. These three peptides are between 37 and 39 amino acids in length and differ only by their amino-terminal phenylalanine (F), alanine (A), and/or leucine residues (LL) [79,80,81]. LL-37, the most common mature form of hCAP18, is found in neutrophils and expressed by many epithelia including the mucosa and integument. Aside from direct antimicrobial mechanisms, LL-37 can also exhibit chemotactic, immunomodulatory and angiogenic effects that are all mediated by antagonistic binding of N-formyl peptide receptor 2 (FPR2), a G-protein coupled receptor. LL-37 was recently shown to inhibit HIV-1 replication using this mechanism, by binding to FPR2 which in turn down-regulated chemokine receptors necessary for HIV-1 entry in primary CD4^+^ T cells [81]. In the FRT, hCAP18 has been immunolocalized to the upper epithelial layers of inflamed ectocervix in a band-like pattern [82]. Under healthy conditions, LL-37 is present in vaginal fluid at concentrations (mid-to-high nanograms/mL) [6] required to act on FPR2 and inhibit HIV-1 replication [81].

Due to structural and functional similarities to several antimicrobial peptides, peptide fragments of histones have also been implicated in the host defense of mucosal surfaces [83,84]. Histones and the related protamines are particularly well-endowed with basic amino acids, and thus their general microbicidal activities are likely related to electrostatic attraction to anionic microbial surfaces. However, the anti-HIV-1 activity of histones appears to be quite distinct from direct membranolytic action. Ubiquitinated histone 1B has been identified as an HIV-resistant factor, possibly regulating viral expression and secretion from CD4^+^ T cells [85]. Although histones are present in the FRT [28], it remains to be determined whether histones have a true antiviral host defense role in this environment.

Larger cationic proteins are also components of human cervicovaginal fluids, and contribute to the collective anti-HIV-1 activity of the FRT. Lysozyme is a cationic 14.6 kDa enzyme whose primary bacteriolytic properties result from cleaving peptidoglycan between N-acetyl muramic acid and N-acetyl-D-glucosamine. Lysozyme also exhibits non-enzymatic properties that likely result from its electrostatically charged surface, which enable the protein to disrupt membranes and activate bacterially derived autolytic enzymes [86,87,88]. Alternative mechanisms of action extend to lysozyme’s ability to inhibit HIV-1. Lysozyme purified from human neutrophils, breast milk, and β-core human chorionic gonadotropin preparations could lower the ability of HIV-1-infected primary T lymphocytes and monocytes to produce virus [89], potentially by directly binding to viral RNA [90]. Peptide fragmentation and activity mapping of human lysozyme revealed that a core nine-residue peptide derived from lysozyme exhibited much greater activity against HIV-1 (IC_50_ 50nM) than the intact protein, and acted to prevent viral entry [91]. While the nonapeptide has not been isolated from biological cells or fluids, its cleavage sites suggest that trypsin or related human proteases could function to liberate this highly active lysozyme-derived anti-HIV-1 peptide *in vivo*.

Cathepsin G, a neutrophil-derived serine protease that is present in human CVF [28], has been reported to bind the HIV-1 envelope protein gp120 [92,93], and can promote HIV-1 infection of macrophages, but not CD4^+^ T lymphocytes [94]. The mechanism of this antiviral activity likely requires G_i_ protein-mediated signal transduction, as treatment of cells with pertussis toxin abrogated the enhancement of HIV-1 infection of macrophages [94]. Interestingly, prolonged exposure of macrophages to cathepsin G suppressed HIV-1 infection, an effect that was neutralized by the addition of serine protease inhibitors [94]. Cathepsin G has also been reported to generate truncated variants of the chemokine RANTES, which exhibited lower binding to CCR5 and reduced antiviral activity [95]. Taken together, these studies suggest a multifactorial role for cathepsin G in enhancing HIV-1 infection.

Lactoferrin is an approximately 78 kDa basic protein, similar in structure and function to the iron-carrier protein transferrin. Lactoferrin can directly and indirectly inhibit HIV-1 by binding to the V3 loop of the HIV-1 envelope glycoprotein gp120, preventing adsorption of the virus to the surface of target cells [96,97]. Although the concentrations of lactoferrin and lysozyme are low in human vaginal fluid (1–13 μg/mL), they are extremely high (100–1000 μg/mL) in the cervical mucus plug [6,60]. Although the anti-HIV-1 activities for both lysozyme and lactoferrin are modest *in vitro*, it may be within the cervical mucus plug where their antiviral host defense properties are best realized.

## 6. Regulation of Cationic Peptides and Proteins in the FRT

Deficiencies in the production of antimicrobial peptides, including activation, release, and/or concentration, have been implicated in the pathogenesis of inflammatory or infectious conditions. Windows of opportunity likely arise in which HIV-1 transmission and infection in the FRT are increased due to mechanisms that enable the virus to subvert innate antiviral host defenses. While multiple components of innate and adaptive immunity are likely involved, this review is centered on how cationic antimicrobial peptides/proteins are modulated, and in the FRT, there are at least three principal strategies in which this regulation occurs: hormonal, microbial, and proteolytic. While earlier studies have suggested that there is little change in the expression of antimicrobial peptides and proteins in the cervicovaginal fluid throughout the menstrual cycle [6], more recently it has been shown that concentrations of HNP1–3, SLPI, lysozyme, lactoferrin, and HBD-2 are all highest during the proliferative phase and to a lesser extent the secretory phase (reviewed in [98]). Oral contraceptives can also alter the expression of a number of peptides and proteins in cervical mucus, including lysozyme [99]. The regulation of defensin HD5, a cationic peptide that enhances HIV-1 infection, is under hormonal control, with maximal expression during the secretory phase [42].

For all studies that measure the concentration of antimicrobial peptides and proteins from lower FRT fluids, the method of collection (lavage, tampon, swab, diaphragm) has a large influence on the amounts and even types of recovered peptides and proteins. This is one reason (of many) why the field has only a coarse understanding of the regulation of antimicrobial peptides in the FRT, as each method of collection has its own merits and detractions. It has yet to be determined which fluid recovery technique would be best suited for the majority of applications and conditions, but it will be important that the field soon adopts a unified approach to reduce inter-study variability.

Sexually transmitted infections of the FRT, such as genital herpes, and microbial-shift conditions, including bacterial vaginosis, have been associated with an increase in the risk of acquiring HIV-1 [100], as well as modulating the expression of cationic peptides and proteins. For example, in HIV-exposed seronegative women in HIV-serodiscordant relationships, the levels of HNP1-3 and LL-37 were directly associated with the partner’s viral load [101]. Selective depletion of cationic peptides and proteins from the cervicovaginal fluids rendered the remaining fractions inactive against HIV-1 [101], supporting the notion that these peptides are major components of innate antiviral host defense. *Neisseria gonorrhoeae*-induced HD5 and HD6 can increase HIV-1 Infectivity [102], which is not surprising since HD-5 is known to promote HIV-1 infection through increasing viral attachment to target cells [43]. Interestingly, in other co-infections, the presence of certain antiviral cationic peptides and proteins suggest roles that run counter to preconceived notions of antiviral defense. LL-37, produced by HSV-2-infected keratinocytes, was reported to upregulate the expression of HIV-1 receptors in monocyte-derived Langerhans cells, enhancing their HIV susceptibility—an effect that could be blocked by inhibiting LL-37 production [103]. While cervicovaginal levels of Trappin-2/elafin are diminished during BV [75], up to 200-fold greater concentration of α-defensins were found in the cervicovaginal fluids of women during frank BV [104]. In a study that collected cervicovaginal fluids from highly HIV-exposed, uninfected Kenyan sex workers, cervicovaginal levels of α-defensins and LL-37 were associated with increased HIV acquisition, which was likely due to sexually transmitted bacterial infections [105].

Proteolytic activation is now recognized as an important mechanism to regulate proteins in the FRT, which modulate HIV-1 infection. In an exciting study by Sorensen and colleagues, following heterosexual intercourse, the human cathelicidin hCAP18 was cleaved into the ALL-38, a peptide that retained complete biological activity as compared to LL-37 [80]. An interesting twist is that the enzyme responsible for this activation was the prostate-derived protease gastricsin, which is present in semen but not in vaginal fluid. Under the slightly basic pH of semen, gastricsin is not able to cleave hCAP18. However, upon incubation with low pH buffers *in vitro*, or contact with the acidic milieu of the vagina *in vivo*, gastricsin was activated and process hCAP18 into ALL-38. Although ALL-38 itself has not been tested against HIV-1, given that all biological tests performed confirm its equivalent potency to LL-37 [80] and that LL-37 can inhibit HIV-1 replication [81], it reasons that gastricsin-mediated activation of hCAP18 represents a novel mechanism to prevent HIV-1 infection following sexual intercourse. As with all cationic antimicrobial peptides and proteins of the cervicovaginal mucosa, it is important to consider that antiviral activity of the FRT is highly dependent on the majority of these molecules acting together, and even slight dysregulation can result in increased susceptibility to HIV-1 transmission and infection [28].
